# Resemblance and Difference of Seedling Metabolic and Transporter Gene Expression in High Tolerance Wheat and Barley Cultivars in Response to Salinity Stress

**DOI:** 10.3390/plants9040519

**Published:** 2020-04-17

**Authors:** Muhammad Zeeshan, Meiqin Lu, Shama Naz, Shafaque Sehar, Fangbin Cao, Feibo Wu

**Affiliations:** 1 Department of Agronomy, College of Agriculture and Biotechnology, Zijingang Campus, Zhejiang University, Hangzhou 310058, China; 11616102@zju.edu.cn (M.Z.); shamaktk@yahoo.com (S.N.); 21516206@zju.edu.cn (S.S.); 2 Australian Grain Technologies, Narrabri, NSW 2390, Australia; meiqin.lu@ausgraintech.com; 3 Jiangsu Co-Innovation Center for Modern Production Technology of Grain Crops, Yangzhou University, Yangzhou 225009, China

**Keywords:** barley, secondary metabolism, salinity, transporter genes, wheat, GSH/GSSG

## Abstract

To elucidate inter-specific similarity and difference of tolerance mechanism against salinity stress between wheat and barley, high tolerant wheat cv. Suntop and sensitive cv. Sunmate and tolerant barley cv. CM72 were hydroponically grown in a greenhouse with 100 mM NaCl. Glutathione, secondary metabolites, and genes associated with Na+ transport, defense, and detoxification were examined to discriminate the species/cultivar difference in response to salinity stress. Suntop and CM72 displayed damage to a lesser extent than in Sunmate. Compared to Sunmate, both Suntop and CM72 recorded lower electrolyte leakage and reactive oxygen species (ROS) production, higher leaf relative water content, and higher activity of PAL (phenylalanine ammonia-lyase), CAD (cinnamyl alcohol dehydrogenase), PPO (polyphenol oxidase), SKDH (shikimate dehydrogenase), and more abundance of their mRNA under salinity stress. The expression of *HKT1, HKT2*, salt overly sensitive (SOS)1, *AKT1,* and *NHX1* was upregulated in CM72 and Suntop, while downregulated in Sunmate. The transcription factor WRKY 10 was significantly induced in Suntop but suppressed in CM72 and Sunmate. Higher oxidized glutathione (GSSG) content was accumulated in cv. CM72 and Sunmate, but increased glutathione (GSH) content and the ratio of GSH/GSSG were observed in leaves and roots of Suntop under salinity stress. In conclusion, glutathione homeostasis and upregulation of the *Ta*WRKY10 transcription factor played a more important role in wheat salt-tolerant cv. Suntop, which was different from barley cv. CM72 tolerance to salinity stress. This new finding could help in developing salinity tolerance in wheat and barley cultivars.

## 1. Introduction

Salinity is one of the most common abiotic stresses constraining crop growth and productivity worldwide. It is estimated that more than 50% of growing land will be affected as a result of mounted salinization in the soil by the year 2050 [1]. The most effective approach to dealing with salinization is developing crop varieties with tolerance. However, lack of genetic resources well-adapted to salt stress and complexity of traits have hampered the progress toward breeding tolerant varieties. In order to overcome these obstacles, therefore, it is important to identify and understand the mechanisms of tolerance for salt stress in existing different genetics resources. 

Redox homeostasis at a cellular level regulated by a multi-system comprised of antioxidants and prooxidants is crucial for plant growth and development under biotic and abiotic stresses [2]. Various mechanisms are involved in the process of enhancing salinity tolerance, for example, the mitigation of toxic radicals scavenging by enzymatic and non-enzymatic antioxidants, compartmentalization of the toxic ions (Na^+^) into vacuole [3], ion uptake and homeostasis, synthesis and modulation of osmolytes and phytohormones and regulatory molecules [4]. Reduced glutathione (GSH), being one of the major elements in the antioxidants defense system, and cellular redox couple (GSH/GSSG (oxidized glutathione)) are important in maintaining cellular redox homeostasis. It takes part in the scavenging of reactive oxygen species (ROS), especially H_2_O_2_, either directly through AsA-GSH (ascorbate-glutathione) cycle and/or play an indirect role in shielding membranes by maintaining zeaxanthin and α-tocopherol in the reduced state [2]. Under stress, GSH also plays an essential role in dumping the ROS and other hazard compounds and controlling the alteration of proteins, usually synthesized by the excessive oxidation of protein thiol groups [5]. Higher GSH and their redox states have been observed in the tissue of tolerant tomato plants upon onset of salinity [6]. An elevated GSH content in salt-tolerant genotypes versus sensitive ones has also been reported in rice [7]. 

Secondary metabolism in plant covers numerous biochemical and physiological aspects of “secondary products” over the evolutionary and functional facets [8]. Plants regulate secondary metabolism upon the onset of stresses so as to indispensably relieve the adverse effect of the stresses by promoting defense and tolerance in plants [9]. Many secondary metabolites are involved in chelating radicals and enhancing the capacity of the plant antioxidant defense mechanism in plant tissues, such as cellulose, flavonoid, and phenols [10]. Secondary metabolites are natural compounds produced by plants and perform a variety of physiological roles. In plants subject to salinization, we hypothesized that high solute concentrations of secondary metabolites could facilitate osmotic adjustment during the stress. In addition, a set of salt stress-related genes is responsible for tolerance mechanism acting via physiological and biochemical process; for example, SOS (salt overly sensitive) acts in essential signaling pathways for ion homeostasis [11], and histidine kinase transporter (HKT) is considered a pivotal gene by regulating the transportation of Na^+^ and K^+^. Similarly, NHX, an intracellular protein, is also involved in K^+^ homeostasis and salt tolerance. Other more on the list are osmoregulatory genes, antioxidant proteins, and transcription factors, such as signal-related protein kinases WRKY and auxin responsive factor (ERF) [12]. 

Wheat (*Triticum aestivum* L.) is a staple food crop for nearly 35% of the world population [13]. Since 1991, China has been one of the top wheat producers in the world. Wheat is also one of the most salt-sensitive species among cereals, while barley (*Hordeum vulgare*) is the most salt-tolerant [14]. Cultivating salt-tolerant wheat varieties is one of the most effective ways to alleviate the negative influence of salt stress [15]. Attempts to improve wheat salt tolerance through the conventional breeding program have resulted in limited success due to the genetic and physiological complexes of the trait and lack of salt-tolerant genetic germplasm. 

Salinity tolerance differs among genotypes in both wheat and barley [16]. Suntop is one of the salt-tolerant wheat cultivars with high grain quality bred and released by the Australian Grain Technologies (AGT) Narrabri breeding program in 2012 and is highly adapted to grow in Australian saline-alkaline fields, with about 15%–30% more yield than the local normal wheat cultivars. Our previous study found that salt tolerance of Suntop was comparable to the salt-tolerant barley *cv*. CM72 [17]. However, the question remains whether it possesses different salt tolerance mechanisms from barley CM72. This study aimed to investigate the mechanisms that confer salinity tolerance in these two species by characterizing species level similarities or differences in glutathione homeostasis, secondary metabolism, and transporters genes upon exposure to salt stress. 

## 2. Results

### 2.1. Relative Water Content, Electrolyte Leakage, and Oxidative Status

**Figure 1:**
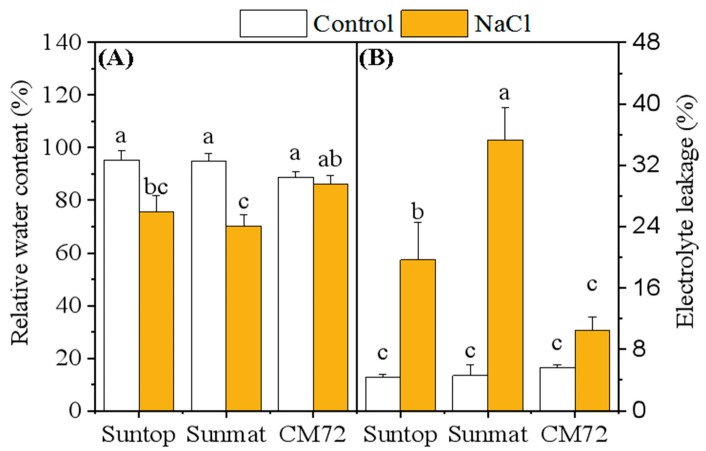
Relative water content (**A**) and electrolyte leakage percentage (**B**) in leaves of two wheat cultivars—Suntop and Sunmate—and one barley *cv*. CM72 after 7 days of exposure to 100 mM NaCl. Control and NaCl represent 0 and 100 mM NaCl, respectively. Values are means ± SD (n = 4). Different letters indicate a significant difference (*p ≤* 0.05) between NaCl treatment and control within each wheat and barley cultivar.

The effect of salinity stress on the relative water content (RWC) and electrolyte leakage (EL) in leaves of wheat and barley plants is presented in Figure 1. RWC was significantly affected by the imposed stress and decreased more significantly in sensitive wheat cultivar Sunmate (–25%) than tolerant cultivar Suntop (–21%) and barley CM72 (–5%). EL from the leaf membrane increased significantly for all tested cultivars under the saline-treated condition, with the largest increases observed in wheat cultivars Sunmate and Suntop. 

**Figure 2:**
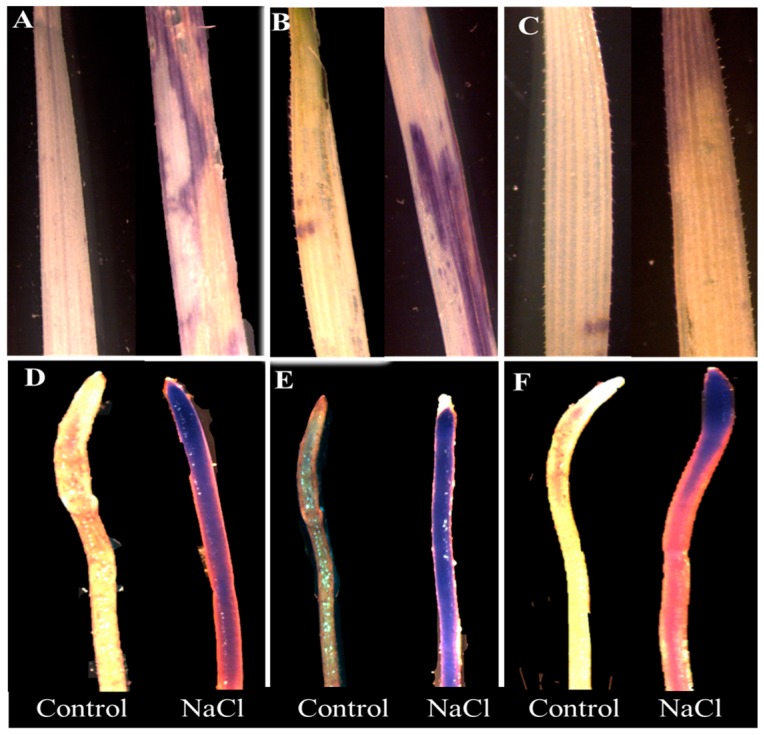
Histochemical detection of H_2_O_2_ by NBT (nitro-blue tetrazolium) staining in leaves (top row) and roots (bottom row) of two wheat cultivars—Suntop (**A**,**D**), Sunmate (**B**,**E**)—and barley cultivar CM72 (**C**,**F**). The samples were collected after 7 days of 100 mM NaCl treatment.

**Figure 3:**
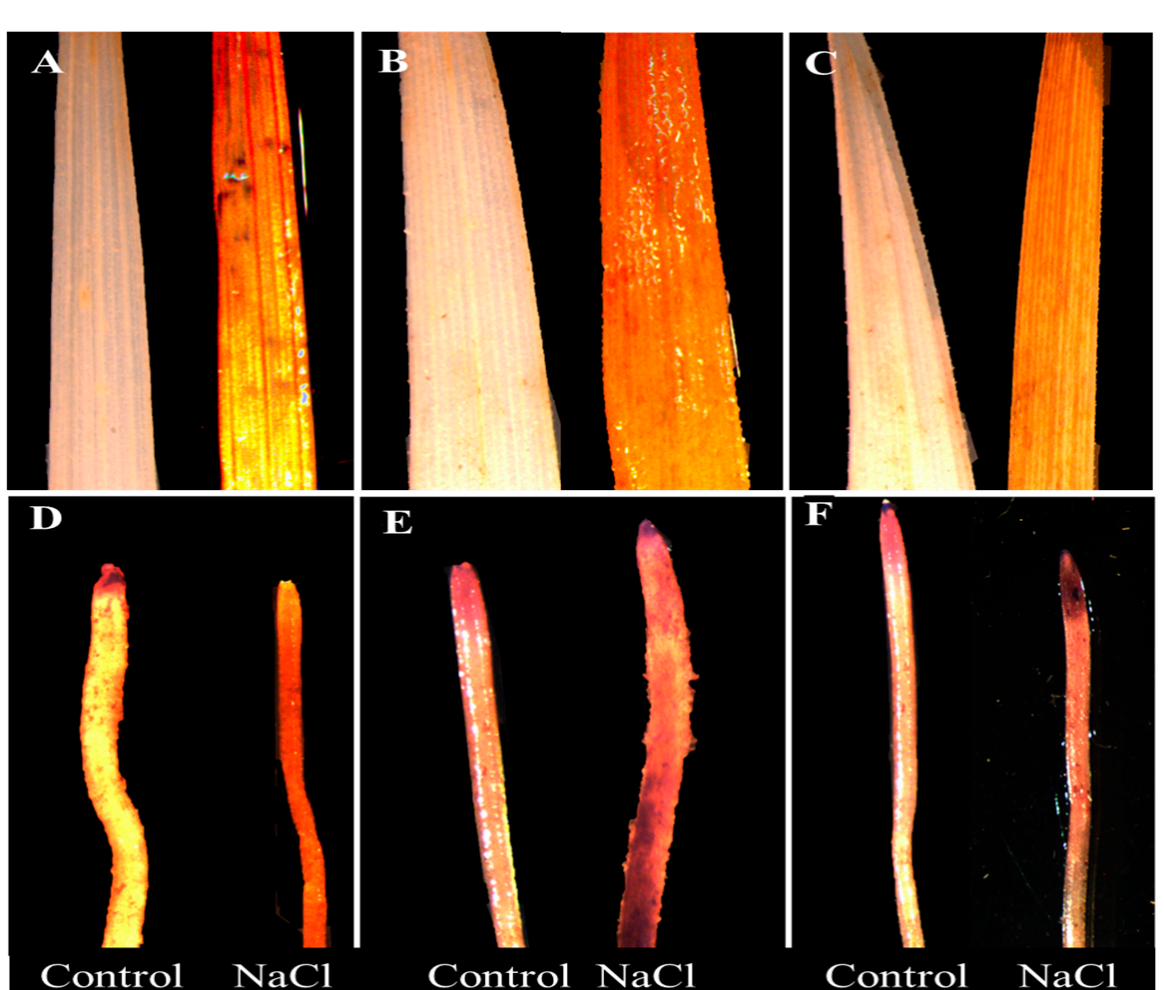
Histochemical detection of H_2_O_2_ by DAB (3,3-diaminobenzidine) staining in leaves (top row) and roots (bottom row) of two wheat cultivars—Suntop (**A**,**D**), Sunmate (**B**,**E**)—and barley cultivar CM72 (**C**,**F**). The samples were collected after 7 days of 100 mM NaCl treatment.

Histochemical staining of leaves and roots revealed a genotypic difference in response to 100 mM NaCl treatment (Figure 2A–F and Figure 3A–F). Untreated plants did not show any visible staining of nitro-blue tetrazolium (NBT) and 3,3-diaminobenzidine (DAB) in the leaves and roots. However, considerable differences were detected among the cultivars under stress. Wheat cultivar Sunmate showed a very dark blue color along the root (Figure 2E), while, in barley *cv*. CM72, blue staining was observed only at the root tip (Figure 2D). Sunmate was the only one with bright and large dark blue spots on leaves. DAB staining, an indicator of higher H_2_O_2_ accumulation, suggested more dark brown color in leaf and root tissues in Sunmate, followed by Suntop and the least in CM72 (Figure 3D–F). 

**Figure 4:**
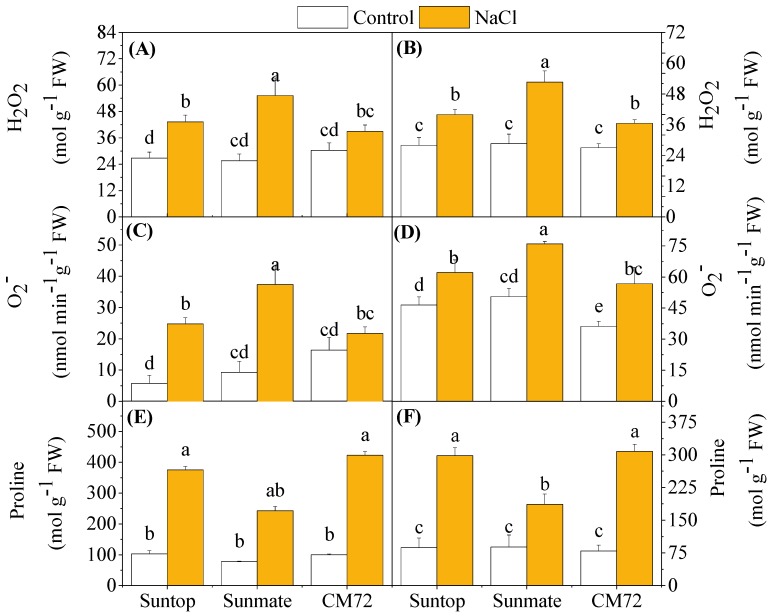
Accumulation of H_2_O_2_ (**A**,**B**), O_2_^–^ (**C**,**D**), and proline content (**E**,**F**) in leaves (left panel) and roots (right penal) of two wheat cultivars—Suntop, Sunmate—and barley cultivar CM72 exposed for 7 days to 100 mM of NaCl. Data are means ± SD (n = 4). Different letters indicate a significant difference (*p* ≤ 0.05) between NaCl treatment and control within the two wheat cultivars and barley *cv*. CM72.

Salinity induced significantly higher accumulation of ROS (H_2_O_2_ and O_2_^–^), but both H_2_O_2_ and O_2_^–^ content in leaves varied with the tested cultivars (Figure 4). Barley cultivar CM72 had the lowest induced accumulation of H_2_O_2_ (+39%, NaCl vs. control), followed by Suntop (+61%) and the highest in Sunmate (+93%) in comparison with their respective controls. In roots, a similar trend was found with H_2_O_2_ content being 84%, 42%, and 35% higher in Sunmate, Suntop, and CM72, respectively, under NaCl stress. An increased O_2_^–^ concentration was also observed for all the tested cultivars under NaCl treatment; however, CM72 appeared to have the least elevated O_2_^–^ in both tissues. Salinity stress increased the proline content in leaves and roots of both species (Figure 4E,F). It elevated much more significantly in both leaves and roots of Suntop and CM72, but the absolute content and increased amount were comparable. 

### 2.2. GSH, Total Phenols, and Flavonoid Content

**Figure 5:**
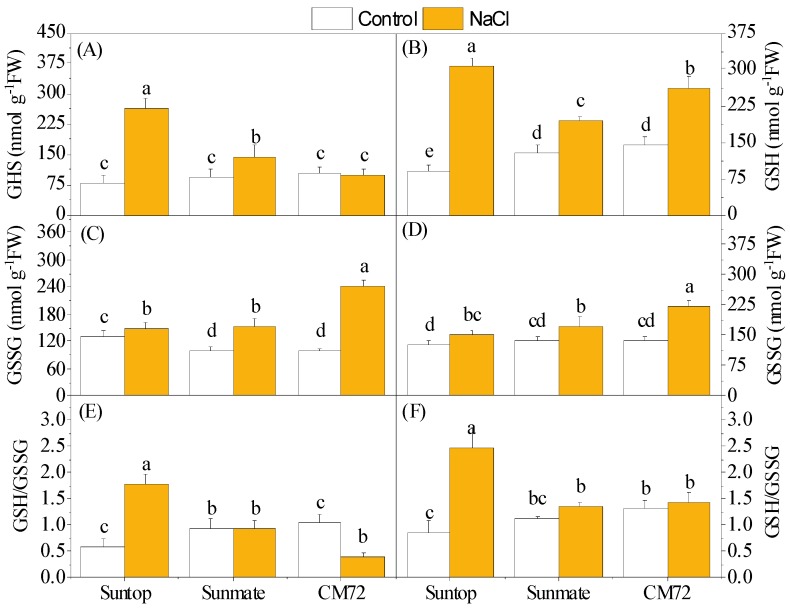
Contents of glutathione (GSH) (**A**,**B**) and oxidized glutathione (GSSG) (**C**,**D**), and the ratio of GSH/GSSG (**E**,**F**) in leaves (left panel) and roots (right penal) of two wheat cultivars—Suntop and Sunmate—and barley cultivar CM72 exposed for 7 days to 100 mM of NaCl. Different letters indicate a significant difference (*p* ≤ 0.05) between NaCl treatment and control within the two wheat cultivars and barley *cv*. CM72.

Upon 100 mM NaCl stress, the GSH contents of the leaf and root were significantly increased except leaves in CM72. The increased amount of GSH content in leaves was in the order Suntop > Sunmate > CM72, and, in roots, it was Suntop > CM72 > Sunmate. GSSG contents in both leaves and roots increased under treatment; however, the increased amount over the control was in the order CM72 > Sunmate > Suntop in both plant tissues (Figure 5A–D). As a result, the increased GSH/GSSG ratio was only recorded in both leaves and roots of Suntop under stress (Figure 5E,F). 

**Figure 6:**
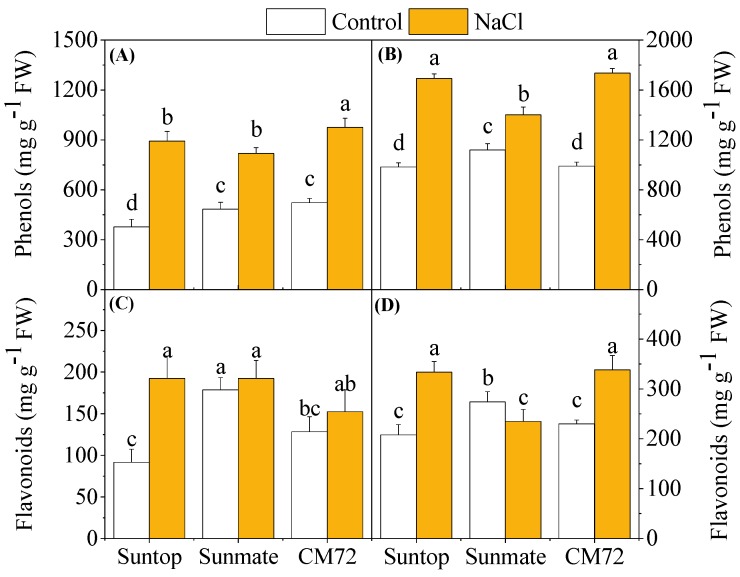
Total phenols content (**A**,**B**) and flavonoids (**C**,**D**) in leaves (left panel) and roots (right penal) of two wheat cultivars—Suntop, Sunmate—and barley cultivar CM72, exposed for 7 days to 100 mM of NaCl. Different letters indicate significant differences (*p* ≤ 0.05) between NaCl treatment and control within the two wheat cultivars and barley *cv*. CM72.

As shown in Figure 6, the content of phenolics and flavonoid was significantly increased (*p* < 0.05) in both roots and leaves of Suntop and CM72 under NaCl stress. Phenolics content in leaves and roots of Sunmate behaved the same as the other cultivars but exhibited less extent of the increase. Flavonoid content remained unchanged in leaves of Sunmate but reduced in its roots under 100 mM NaCl. 

### 2.3. Phenolic Metabolism-Related Enzymes

**Figure 7:**
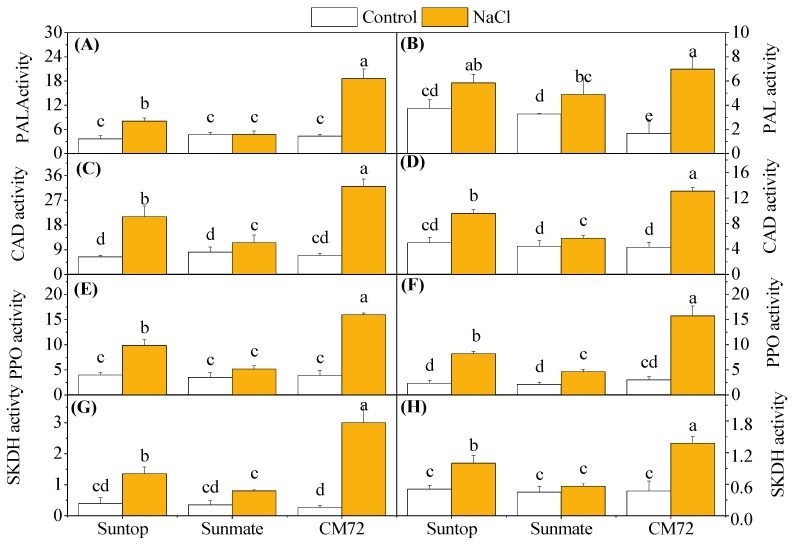
Activities of secondary metabolism-related enzymes: phenylalanine ammonia-lyase (PAL; **A**,**B**) (Unit mg^–1^ protein), cinnamyl alcohol dehydrogenase (CAD; **C**,**D**) (mol mg^–1^ protein); polyphenol peroxidase (PPO; **E**,**F**) (Unit mg^–1^ protein), and shikimate dehydrogenase (SKDH; **G**,**H**) (mol mg^–1^ protein) in leaves (left panel) and roots (right penal) of two wheat cultivars—Suntop, Sunmate—and barley cultivar CM72, exposed for 7 days to 100 mM of NaCl. Different letters indicate significant differences (*p* ≤ 0.05) between NaCl treatment and control within the two wheat cultivars and barley *cv*. CM72.

Figure 7A–H shows PAL, PPO, CAD, and SKDH contents in the leaves and roots of wheat and barley plants when grown with and without 100 mM NaCl. The activity of PAL in leaves and roots of CM72 was significantly increased under 100 mM NaCl. This also occurred in wheat cultivar Suntop, despite the extent of the increase being smaller than that observed in CM72. In Sunmate, the activity of PAL was increased only in roots under NaCl treatment (Figure 7A,B).

Salinity induced significantly higher CAD activity in leaves and roots of all cultivars, and the increased amount was in the order CM72 > Suntop > Sunmate (Figure 7C,D); in detail, it was 4.2-, 3.3-, and 1.9- times increase in leaves and 3.1-, 1.9-, and 1.3 times increase in roots for the above cultivars, when compared to their respective controls.

Compared with control, PPO activity also significantly increased under salinity stress in the leaves and roots of both species, except in the leaves of Sunmate (Figure 7E,F). Again, the largest boost of PPO activity was observed in leaves (3.7-fold) and roots (5.2-fold) of CM72, followed by Suntop (2.5- and 3.5-folds), and the least in Sunmate. However, a small increase was observed in the tissues of the Sunmate. 

SKDH activity was also measured in both roots and leaves of wheat and barley cultivars under salinity stress (Figure 7G,H). CM72 had the highest SKDH activity in both tissues when exposed to the treatment, while Sunmate only showed minor response; the treatment effect on Suntop was intermediate. The increased folds for SKDH activity in leaves and roots were 9.8 and 2.9 in CM72, 3.4 and 2.0 in Suntop, and 1.8 and 1.2 in Sunmate, respectively. 

### 2.4. Relative Expression of Scavengers and Defense-Related Genes

**Figure 8:**
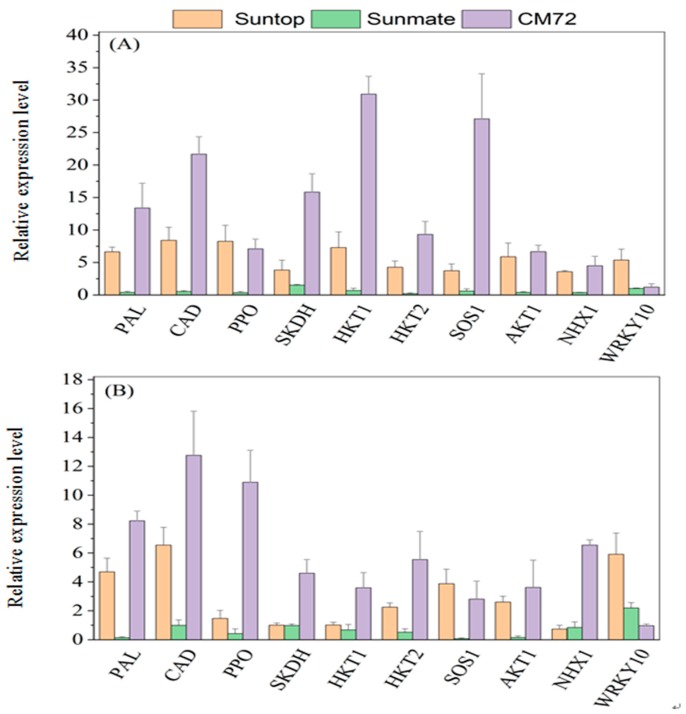
Relative expression levels (100 mM NaCl vs. control) of secondary metabolites-related genes, transporter and sequestration genes, and transcription factors in leaves (**A**) and roots (**B**) of Suntop, Sunmate, and CM72 under 100 mM NaCl. Seedlings were exposed to 100 mM salinity (NaCl) treatments for 7 days. Data are means ± SD (n = 3).

The expression of the genes related to the defense and detoxification of harmful effect of high salinity in wheat and barley plants are given in Figure 8A,B. In leaves, transcript abundance for PAL, PPO, CAD, and SKDH exhibited highest in barley *cv*. CM72 with 10.1-, 6.8-, 42-, and 16.8-fold increase, followed with wheat tolerant *cv*. Suntop (6.6-, 8-, 4.1-, and 3.6-fold increase) and the least in wheat sensitive *cv*. Sunmate being (0.21-, 0.33-, 0.26-, and 1.5-fold increase) under the treatment. Root transcripts abundance for these four genes was slightly different from their expression in leaves; for example, it is notable that the abundance of these transcripts was much more elevated in CM72 than two wheat cultivars (Figure 8B).

### 2.5. Relative Expression of Transporter Genes and Transcription Factor

In comparison to control, the expression of *HKT1, HKT2, SOS1, AKT1,* and *NHX1* in leaves and roots was significantly upregulated in barley *cv*. CM72 and wheat *cv*. Suntop under treatment, while downregulated in wheat *cv*. Sunmate. For the expression of *HKT1, HKT2, SOS1, AKT1,* and *NHX1*, in leaves, it recorded 30.8-, 10.2-, 26.5-, 6.2-, and 4.3-fold increase (NaCl vs. control) in CM72 and 7.0-, 7.6-, 3.6-, 4.5-, and 3.6-fold increase in Suntop, while only a small decrease in Sunmate (Figure 8A). In roots, the increased expression of these genes remained higher in CM72 (3.5, 4.7, 2.6, 3.2, and 6.5 times increase); however, in Suntop and Sunmate, the increased expression was observed but small except *SOS1* and *HKT2* (Figure 8B).

The induced expression of *WRKY10* in the wheat *cv*. Suntop was higher in both leaves and roots, while, in wheat *cv*. Sunmate and CM72, it was only slightly increased with no statistical difference from the control (Figure 8A,B).

## 3. Discussion

Plants adapt two main approaches to deal with salinity stress, such as Na^+^ exclusion and Na^+^ accumulation at tissue level [18]. However, the failure to do so ultimately stimulates the oxidative burst and then causes extensive cellular damage [19]. Some crops, including barley, deploy a strong defensive mechanism, while others, like wheat, adapt a weak and/or moderate defense mechanism to avoid the accumulation of ROS and relieve oxidative burst at the cellular level. Although they belong to the same family, the former is more salt-tolerant [20]. In this study, we attempted to understand similarities and differences between these two species in their response to salinity stresses using a range of biochemical and molecular parameters or biomarkers. 

Salinity induced significant changes in relative water content, endorsing that the two species were compromised to some extent by NaCl stress. High salinity at root medium imposed low osmotic potential and would thermodynamically hamper water uptake by roots. This is a likely reason for reduced relative water content in leaves [21]. In this study, the highest RWC decline in wheat sensitive *cv*. Sunmate than tolerant *cv*. Suntop and barley *cv*. CM72 indicated that it could be one of the attributes, differentiating tolerant and sensitive cultivars. Our result also demonstrated that sensitive cultivar Sunmate rather than tolerant ones Suntop and CM72 showed a higher electrolyte leakage under NaCl condition. This agreed with the previous findings that the low pass out of EL is co-related with salinity tolerance in wheat [22] and barley [23], and, therefore, maintenance of membrane integrity and stability is an important factor behind salt tolerance in plants. 

The stressful condition can promote ROS production, such as, hydroxyl radicals (OH^−^), superoxide radicals (O_2_^−^), and hydrogen peroxide (H_2_O_2_), which directly alters the redox homeostasis by causing oxidative damage to biomolecules, such as proteins, nucleic acids, and phospholipids [24]. Leaves of barley *cv*. CM72 showed non-significantly induced accumulation of H_2_O_2_ and O_2_^–^ over the control. This was further supported by *in vivo* DAB and NBT staining, all of which suggested reduced and low accumulation of H_2_O_2_ and O_2_^–^ in both tissues of CM72 plants. Interestingly, the absolute content of two ROS in leaves and roots of wheat tolerant cultivar Suntop was equivalent to that of barley cultivar CM72 under treatment; however, they were increased more significantly over the control (Figure 4A–D). In parallel to that, the increased ROS response was much higher in wheat sensitive cultivar, Sunmate. Therefore, stimulated production of ROS in wheat cultivars is common and must cause oxidative stress and ultimately stunt the growth of wheat plants like what observed in Sunmate in this study.

Glutathione is the low molecular weight antioxidative metabolite, plays important roles in defense against oxidative stress in plants, and remains in focus for engineering to develop resistant plants against various stresses [2]. Higher content of GSH helps in balancing the GSH/GSSG ratio and acts as a redox buffer by participating in ROS scavenging as well as signaling under high-stress conditions [25]. In our study, higher accumulation of GSH content and GSH/GSSG ratio under salinity stress was detected in the leaves and roots of Suntop over CM72 and Sunmate. It seemed that higher GSH content in Suntop conferred plant ability to acclimatize under high NaCl concentrations. Considering another role of GSH mentioned before, this evidence might suggest that Suntop was able to alleviate the damage caused by hyperproduction of ROS and also partially explain why Suntop suffered less oxidative damages than Sunmate. However, no or lower elevation in the GSH content under salinity stress in both tissues of barley cultivar CM72 seemed counter-intuitive in this study (Figure 5A,B). 

Under stress treatment, plants start overproduction of proline to maintain osmotic balance or cell turgor [26], stabilize membranes, limit the rate of electrolyte leakage, and keep reactive oxygen species concentration under normal condition, thereby alleviating oxidative burst in plants [27]. In the present study, a remarkable increase in the proline content in CM72 and Suntop over Sunmate might suggest its effective role as ROS scavengers. This, together with the aforementioned higher relative water content in leaves and minimal electrolyte leakage, would enhance the tolerance of these two cultivars against salinity stress. Furthermore, an increase in TPC and flavonoids content was observed in saline-treated plants in all tissues of the tested cultivars. However, neither TPC nor flavonoid content showed a clear difference between Suntop and CM72 cultivar under the saline treatment. Similar to our finding, Ma et al. (2014) [28] also reported the elevated content of total phenols and flavonoids in wheat tissue under drought conditions.

Salinity stress induced the phenolic compound via the phenylpropanoid pathway, which is generally initiated by PAL activity [29] and the identified caffeic acid derivatives [30]. In our experiment, barley cultivar CM72 showed the elevated activity of PAL, CAD enzymes, and their mRNA abundance upon salinity stress when compared with wheat cultivars, which, in turn, explained the increased accumulation of total phenol and flavonoid content (Figure 7A–D). Elevated production of phenolic metabolites, including lignin, is a universal response to environmental stresses, including salinity, and helps plants to relieve the stressful moment [31]. This molecule is a highly specific biomarker for lignification and catalyzing the conversion of cinnamyl aldehydes to cinnamyl alcohols [32]. Both PAL and CAD molecules are involved in the lignin precursor production [33], and considerable overlapping expression pattern is expected, which was the case for tolerant cultivars in our study. So, highly increased activities of PAL and CAD in barley CM72 clearly suggested their involvement in the tolerance mechanism. In the tolerant wheat cultivar, Suntop, they played a similar role but not as strong as what was observed in barley CM72 (Figure 7 and Figure 8).

The increased PPO activity, together with high relative expression of PPO gene, in leaves and roots tissues of barley *cv*. CM72 and wheat *cv*. Suntop was detected under salinity stress. The relationship between salt tolerance and accumulation of PPO was found, as salt-tolerant *cv*. CM72 and Suntop and salt-sensitive *cv*. Sunmate were significantly different in different concentrations of PPO. This finding supported that plants with accumulated polyphenol oxidase (PPO) contents under stress treatment could oxidase and degrade toxic phenolic compounds accumulated during the salt stress [34]. Demir and Kocaçalişkan (2001) [35] also found increased PPO content in bean seedling, while Agarwal and Pandey (2004) [36] reported preliminarily that elevated polyphenol oxidase (PPO) concentration alleviated the accumulation of phenol compound in the senna seedling against NaCl. Furthermore, SKDH activity help plant to convert simple carbohydrates to the aromatic amino acid. In this study, the highest increase of activity of SKDH under saline stress in the roots and leaves was detected in CM72, but the increase in wheat cultivars was very small. Consistent with this finding, Ahmad et al. (2015) [37] reported a boosted SKDH enzyme activity in barley genotypes and Tesfaye et al. (2019) [38] in wheat genotypes under combined stress of drought and salinity. 

Some sodium transport pathways, namely, high-affinity K^+^ transporter (*HKT1, HKT2*), salt overlay sensitive (*SOS1*), *AKT1,* and *NHX1,* actively participate in Na^+^ ion exclusion, Na^+^ extrusion by plasma membrane Na^+^/H^+^ exchange transporters. Additionally, restricting Na+ uptake and/or enhancing cytoplasmic potassium (K^+^) levels relative to Na^+^ encourage to increase Na^+^ tolerance in plants [39]. Na^+^ and K^+^ are co-transported from roots to leaves via *HKT* pathways. We investigated the *HKT* family transcript abundance to understand the differences between wheat and barley. We found a salt-induced up-expression in *HKT1* and *HKT2* genes in both tissues of CM72 and Suntop, confirming their role in the sodium retrieval from the xylem vessels, thus, restricting the Na^+^ moment towards shoots [40]. The salt-induced up-expression of these genes in the roots of CM72 and Suntop can help to restrict the moment of Na^+^ towards the sensitive photosynthesis tissue; in the meantime, it may also reduce the amount of Na^+^ loaded into the xylem so as to maintain the better performance of plants under salinity stress. In our previous study, we found a higher concentration of Na^+^ content in shoots of wheat sensitive *cv*. Sunmate compared to the tolerant *cv*. Suntop and barley *cv*. CM72, suggesting that Sunmate failed to some extent to restrict the accumulation of Na^+^ in sensitive tissue like leaves or leaf canopy [17]. Besides, we also investigated plasma membrane Na^+^/H^+^ antiporter, namely, *SOS1*, a long-distance Na^+^ transport controller [41] that involves in transporting Na^+^ into extracellular space after Na^+^ enters the cell [42]. We found that the expression level of *SOS1*-like Na^+^/H^+^ exchanger in the leaves and roots of *cv*. Sunmate was suppressed while induced in wheat *cv*. Suntop and barley *cv*. CM72. Shi et al. (2003) [43] interpreted that the increased activity of *AtSOS1* improved salt tolerance in transgenic Arabidopsis, while Zhu et al. (2016) [44] concluded that Na^+^ exclusion and H^+^ uptake in the root epidermis were reduced after the SOS1 exchanger activity was suppressed under saline conditions. 

The inward rectifier K^+^ channel (*AKT1*) is considered an important pathway for the uptake of K^+^ in root cell [45], but, interestingly, under the saline condition, *AKT1* pathways are more selective for Na^+^ over K^+^ [46]. The evaluation of the relative expression of *TaAKT1* and *HvAKT1* transcripts in leaves and roots of wheat and barley *cv*. under 100 mM NaCl for seven days exhibited differences in gene expression patterns (Figure 8A,B). Salinity stress induced an increased expression in the *TaAKT1* and *HvAKT1* genes in CM72 and Suntop, while suppressed expression was detected for the *TaAKT1* gene in both tissues of Sunmate. The result demonstrated that barley *cv*. CM72 (Na^+^ excluding cultivar) didn’t import Na^+^ through the *AKT1* channel, and 100 mM NaCl concentration might be within the bearable range. However, wheat *cv*. Suntop, to some extent, and Summate to higher extent took up the amount of Na^+^ through inward rectifying K^+^ channels. Previous studies have shown the wide spectrum of differences in the relative expression of *AKT1* genes under the saline condition in both helophyte and glycophyte species [47,48]. For example, suppressed regulation of *AKT1* gene has been observed in common ice plant, rice, and Arabidopsis in the presence of excessive Na^+^ [47,48]. 

The salt-induced up-expression of wheat and barley vacuolar Na^+^/H^+^ antiporter *TaNHX1* in Suntop and *HvNHX1* in CM72 plants conferred their salt tolerance. The wheat *cv*. Sunmate deprived the expression of *NHX1* genes and resulted in a higher accumulation of Na^+^ at its tissues. Our previous study showed a higher accumulation of Na^+^ in roots and shoots in the wheat and barley when compared to the control. This is likely to be the consequence of the activity of up-expressed vacuolar Na^+^/H^+^ antiporter *NHX1* under stress. The plant’s ability to Na^+^ compartmentalization into vacuoles provides an efficient mechanism to deal with the toxic effect of Na^+^ in the cell cytosol level [49]. Similarly, Gaxiola et al. (2001) [50], in their prior studies, indicated that overexpression of the vacuolar Na^+^/H^+^ antiporter (*AtNHX*1) in Arabidopsis and tomato plants provided enhanced cellular-level tolerance to salinity by increasing the capacity to accumulate cations (Na^+^ and/or K^+^) in the vacuole. 

Recently, attention has been diverted to the WRKY transcription factors controlling plant growth and development under biotic and abiotic stresses [51]. Our quantitative PCR result demonstrated that *Ta* WRKY10 and *Hv*WRKY10 were induced by salinity in the leaves and roots of wheat and barley after 7 days of NaCl treatment. Increased expression of WRKY10 was noted in the wheat tolerant cultivar Suntop over Sunmate and CM72. Surprisingly, the *Hv*WRKY10 transcript abundance in CM72 was no more than that observed in control, although barley as a whole and cultivar CM72, in particular, are considered to have good salinity tolerance. The lower expression of *Hv*WRKY10 might suggest that 100 mM NaCl treatment might not be enough to trigger the expression of this transcription factor (TFs) and/or *Hv*WRKY10 transcription factors might only response to seedling stage salt stress, but this postulation needs to be further examined. In contrast, semi-quantitative RT PCR analysis of *Ta* WRKY10 transcription factor (TFs) using wheat cultivars showed upregulation under salinity stress, which was consistent with the result obtained by Li et al. (2013) [52], who showed that *Ta*WRKY10 was induced under the polyethylene glycol (PEG), NaCl, and mannitol stress conditions in the tolerant genotype of wheat. 

## 4. Materials and Methods

### 4.1. Plant Material and Experimental Design

A hydroponic experiment was conducted in a greenhouse at Zijingang Campus, Zhejiang University, Hangzhou China in 2018. We used 2 wheat *cv*. Suntop and Sunmate, and one salt-tolerant barley *cv*. CM72. Suntop and Sunmate are two high yielding Australian Prime Hard varieties bred by Australian Grain Technologies. Although derived from the same cross, Suntop and Sunmate differ significantly in salt tolerance and sensitivity, respectively. Seeds were washed and surfaced sterilized with 3% H_2_O_2_ for 10 min and germinated on moist sand. After 10 days of germination (two-leaf stage), the uniform seedlings were transferred to 5 L container (191 mm diameter and 210 mm height) filled up with 4.5 L basal nutrient solution [53]. The container was covered with a polystyrol-plate with 9 evenly spaced holes (2 plants per hole) and placed in a greenhouse with natural light and a temperature of 20 ± 2 °C/day and 15 ± 2 °C/night. On the 10^th^ d after transplanting, salinity (as NaCl) was added to the corresponding containers to form 2 treatments: basal nutrient solution (control) and 100 mM NaCl (NaCl). A randomized complete block design was used with four biological replications containing 16 plants for each replication per genotype. The nutrient solution was renewed every 4 d, and the air pump was used to provide continuous aeration to the plants. Solution pH was frequently adjusted to 5.8 with HCl or NaOH as required. Plants were sampled after 7 d of salinity treatment, and plants were separated in roots and leaves and washed with distilled water and then stored at −80 °C for further biochemical and molecular determination.

### 4.2. Relative Water Content and Electrolyte Leakage

The second fully expanded leaf was chosen for the measurement of relative water content (RWC). Leaf was cut from the base of lamina and immediately weighed for the fresh weight (FW); the same leaf was dipped in distilled water for 18 h at room temperature to record the turgid weight (TW). Later, the leaf was drying at 80 °C for 3 d for the measurement of dry weight (DW). Relative water content (RWC) was estimated by the formula, RWC = (FW-DW)/ (TW-DW) × 100. 

The membrane permeability of the leaves was calculated by electrolyte leakage using electrical conductivity meter, according to the following formula [54]:

$$EC=\frac{EC1}{EC2}\times 100$$

### 4.3. Determination of Total Phenol and Flavonoids Content

Total phenol content (TPC) assay was carried out according to Singleton et al. (1999) [55] and based on the Folin–Ciocalteu reagent reduction. For this aliquot (0.125 mL), 10-fold diluted roots and leaves samples of extract were added to 0.5 mL of deionized water and 0.125 mL of the Folin-Ciocalteu reagent. The mixture was shaken and allowed to stand for 6 min, before adding 1.25 mL of 7% Na_2_CO_3_ solution. The solution was then diluted with deionized water to a final volume of 3 mL and mixed thoroughly. After incubation for 90 min at 23 °C, the absorbance versus prepared blank was read at 760 nm. 

Flavonoid content assay was followed according to the method described in [56]. For this, 1 mL of roots and leaves supernatant (0.1 g/mL) was diluted with 4 mL of water and was mixed with 0.3 mL of NaNO_2_ (5% w/v). After 5 min, 0.3 mL of AlCl_3_ (10% w/v) was added, followed by the addition of 2 mL of NaOH (1 M) six minutes later. The reaction volume was increased up to 10 mL by adding 2.4 mL distilled water, and the sample was incubated at room temperature (RT) for 15 min. The absorbance was measured at 510 nm using a spectrophotometer. Total soluble protein was calculated by spectrophotometer using bovine serum albumin as a protein standard [57]. 

### 4.4. Measurement of Secondary Metabolite Enzyme Activity

Secondary metabolites-related enzymes were measured using the crude extract of roots. Phenylalanine ammonia-lyase (PAL, EC 4.3.1.5) activity was assayed following the modified procedure of Dai et al. (2006) [58]. Fresh samples of leaves and roots (0.2 g) were ground and homogenized in the combined mixture of 5 mL (100 mM) of borate buffer, 0.05 g of polyvinyl propyl (PVP), and 5 mM of β-mercaptoethanol having pH 8.8. The supernatant was collected for PAL assay after homogenates were centrifuged at 15,000 g for 15 min. The assay reaction mixture included 0.5 mL of supernatant from leaves and roots source, 2 mL of the same concentration of sodium borate buffer, and 0.5 mL (3 mM) of L-phenylalanine. The reaction mixture was incubated for 1 h at 37 °C. The absorbance was recorded at A290 using a spectrophotometer (SPECTRO star Nano, BMG LEBTECH, Germany), based on the yield of cinnamic acid; control was set without L-phenylalanine. 

Polyphenol peroxidase (PPO, EC 1.14.18.1) activity assay was performed according to the method by Ruiz et al. (1999) [59]. The reaction mixture included 0.1 mL supernatant extract, 0.9 mL sodium acetate buffer (pH 5.0), and 0.1 mL catechol. The increase in absorbance was calculated at 390 nm by a spectrophotometer. 

Guidi et al. (2005) [60] method was followed for the determination of cinnamyl alcohol dehydrogenase (CAD, EC 1.1.1.195) activity. The increase in absorbance was determined at 400 nm after coniferyl alcohol was appropriately oxidized to conifer aldehyde for 5 min at 30 °C. The reaction mixture contained 100 mL enzyme extract, 100 mM coniferyl alcohol, 100 mM Tris-HCl (pH 8.8), and 2 mM nicotinamide adenine dinucleotide phosphate (NADP). The shikimate dehydrogenase (SKDH, EC 1.1.1.25) assay was performed according to the method of Díaz et al. (2001) [61].

### 4.5. Histochemical Staining by DAB and NBT and Assay of H_2_O_2_ and O_2_^–^

Histochemical staining of H_2_O_2_ and O_2_^–^ in both leaves and roots tissues was performed using 3,3-diaminobenzidine (DAB) and nitroblue tetrazolium chloride (NBT), respectively, following the modified method of Chen et al. (2010) [62]. For the determination of H_2_O_2_, plant samples (leaf and roots) were weighed (0.2 g), ground in liquid nitrogen, and the crude enzyme was extracted using 2 mL (0.1%, w/v) trichloroacetic acid (TCA). H_2_O_2_ activity was observed at 390 nm wavelength following the H_2_O_2_ activity assay kit A064-1 (Jiancheng Bio Co., Nanjing, China). Superoxide radical O_2_^–^ was measured according to the modified procedure of Jiang and Zhang (2001) [63]. Briefly, leaf and Root samples were homogenized in the 65 mM of potassium phosphate buffer (pH 7.8). The assay mixture comprised of 0.9 mL potassium phosphate buffer (65 mM) and 0.1 mL hydroxylamine hydrochloride (17 mM), 1 mL naphthylamine (7 mM), and 1 mL of sulphanilamide (17 mM) and n-butanol. The absorbance was calculated at 530 nm using a spectrophotometer (SPECTRO star Nano, BMG LEBTECH, Germany), and a standard curve was developed for the calculation of O_2_^–^ concentration in the samples.

### 4.6. Quantification of Proline and Glutathione and Reduced Glutathione

Proline assay was carried out according to Bates et al. (1973) [64]. For example, 0.2 g fresh weight was homogenized in 2 mL of 3% aqueous sulfosalicylic acid and centrifuged at 10,000 rpm for 30 min. The supernatant was decanted, and the pellet was washed twice with 3% aqueous sulfosalicylic acid. The supernatants were pooled, and the proline content was measured using ninhydrin reagent and toluene extraction. Its OD values were measured at 520 nm spectrophotometrically using toluene as a blank, and its concentration was estimated using a standard curve.

Total glutathione, oxidized glutathione (GSSG), and reduced glutathione (GSH) were measured following the recycled method of Tietze (1969) [65] using the 5,5′-dithio-bis (2-nitrobenzoic acid) (DNTB) and glutathione reductase (GR). For instance, samples (0.3 g) were homogenized with 5 mL of 10% (w/v) trichloroacetic acid (TCA) and centrifuged at 15,000 g for 15 min. The oxidized glutathione (GSSG) was measured by adding 120 μL supernatant in 10 μL of 2-vinylpyridine, followed by adding 20 μL of 50% (v/v) triethanolamine. The solution was vortexed for 30 s and incubated for 25 min at 25 °C. The values of total GSH and GSSG for each sample were calculated from the standard curve generated with known amounts of GSSG. The level of GSH for each sample was obtained by subtracting the GSSG level from the total GSH.

### 4.7. Gene Expression Analysis by qRT PCR

Total RNA was extracted from both root and leaves tissue of control and treated plant samples using the total RNA extraction kit (Takara, Dalian, China) according to the recommended protocol of manufacturers. RNA abundance was quantified with Nanodrop, while purity was confirmed on 1% agarose gel. For real-time RT-PCR analysis, 500 μg of total RNA was reverse transcribed by using the primer Script RT reagent Kit (Takara, Dalian, China). The cDNA samples were assayed by quantitative real-time PCR (qRT-PCR) in the Lightcycler480 (Roche International Diagnostics Systems, Switzerland) as follows: initial denaturation at 95 °C for 30 s, 40 cycles for denaturation and annealing (95 °C for 5 s and 60 °C for 30 s, respectively), followed by steps for Melt-Curve analysis (60−95 °C). The assay was performed using SYBR Green PCR Mastermix (Applied Biosystem, Hangzhou, China). List of gene-specific primers’ sequence of both wheat and barley plants are given in supplementary Table S1. 2^-ΔΔCt^ method was used to compare the transcript levels between different samples [66], as follows:

$$2^{-\Delta \Delta \mathrm{Ct}\;}=\left(Ct_{gene}-Ct_{endo\;Ref}\right)\;\mathrm{treated}\;\mathrm{sample}\;-\left(Ct_{gene}-Ct_{endo\;Ref}\right)non\;treated\;sample$$

### 4.8. Statistical Analysis

Pooled data were subject to ANOVA analysis using SPSS software (Statistical Program for Social Science), following Duncan’s multiple range tests (DMRT) to distinguish between the means at *p* ≤ 0.05. Origin Pro (Version 8.0, Origin lab corporation, Wellesley Hills, Wellesley, MA, USA) was used to prepare graphs.

## 5. Conclusions

This study revealed the similarity and difference in the mechanisms of higher tolerant wheat *cv*. Suntop and barley *cv*. CM72 under salinity stress regime. It was concluded that a component of glutathione homeostasis and upregulation of the *Ta*WRKY10 transcription factor were tolerant mechanisms in *cv*. Suntop, while CM72 adapted secondary metabolism and effectively damped Na^+^ either by compartmentalization and/or restricting Na^+^ uptake or unloading from xylem as the mechanisms.

## Supplementary Materials

The following information is available online at https://www.mdpi.com/2223-7747/9/4/519/s1. Table S1: List of primers used in qRT-PCR of wheat and barley genes.
