# Supplementary material for: Resemblance and Difference of Seedling Metabolic and Transporter Gene Expression in High Tolerance Wheat and Barley Cultivars in Response to Salinity Stress
Source: Plants (Basel). 2020 Apr 17;9(4):519. doi: 10.3390/plants9040519 (PMC7238149; doi:10.3390/plants9040519)
Supplement: Supplementary file 1 [file plants-09-00519-s001.pdf]

Supplementary Table S1. List of primers used in qRT-PCR of wheat and barley genes

	Gene	Primers type	Primer Sequence (5´-3´)	Length
**Wheat**	*TaPAL*	F	CGAGCACGGGTTCTTTGAG	19
		R	CTCAGCAAGGACGGACAGA	19
	TaPPO	F	CCATACGATTCGATTGACCTGT	22
		R	GAGAAGGACCACAAGCCGTA	20
	TaCAD	F	GCACGAGGCAAAGGCAAG	18
		R	TCGACGCTGCCCATTCTC	18
	TaSKDH	F	GCGCCTTGTCGAATTTGGAG	19
		R	AGAGTGCTGACTAGCCGGA	19
	TaHKT1	F	CTGTCGCTCTTCTGCGCCAT	20
		R	TTATACTATCCTCCATGCCT	20
	TaHKT2	F	GATCCACTCAACTTCTCCAC	20
		R	TCATACTTTCCAGGATTTAC	20
	TaSOS1	F	GTTGTCGGTGAGGTCGGAGGG	21
		R	TCATCTTCTCCTACCGCCCTGC	22
	TaAKT1	F	CGGATAATGCCGTGAATG	18
		R	TCTGTATCGGCGGTCGTA	18
	TaNHX1	F	CTCAAGGGTGACTACCAAGCA	21
		R	CCAATGCATCCATCCCGAC	20
	TaWRKY10	F	GGAGCGTCGTCTTCCCTATG	20
		R	CATAGCACCGATCCCTCCTC	20
	TaACTIN	F	GTGCCCATTTACGAAGGATA	20
		R	GAAGACTCCATGCCGATCAT	20
**Barley**	*HvPAL*	F	CCCAAGTTTGGACTATGGCT	20
		R	ATCTTGGTTGTGCTGCTCTG	20
	HvPPO	F	TCTACAACGAGAGGCGTGAC	20
		R	GGTCGATCAGCTGGTCTCTT	20
	HvCAD	F	AAGAGACAGGAGGCTTTGGA	20
		R	GGTAAGGCTCAAGAGGGTGA	20
	HvSKDH	F	TCTTGCAAACACAACAGCAA	20
		R	CACATTCTGCAGCTTCCCTA	20
	HvHKT1	F	GGGTTCGATGTGCAAAGCTC	20
		R	CTCCCGTATGTTCTCCGCATG	20
	HvHKT2	F	GTTCATGACTGCATGACACTTC	20
		R	CCCTCTCTTGGCTTTCGTGT	22
	HvSOS1	F	GGTTCCTTCAGATGAGCAGGTT	22
		R	ACTTTTTAGCTTCCTCCCCAGC	22
	HvAKT1	F	ACTTTGCCGTGATCCATATC	20
		R	CTGGATTCTTGATCATGAGC	21
	HvNHX1	F	ACCGTGAGGTTGCGCTTATG	20
		R	CAGCAATGAAGGACAAGGTTGC	22
	HvWRKY10	F	GGGGAAGCAGCAGGTAGA	18
		R	TGCGGTGGTTGAGGATAG	18
	HvActin	F	GACTCTGGTGATGGTGTCAGC	21
		R	GGCTGGAAGAGGACCTCA	18
